# Supplementary material for: Multi-modality detection of SARS-CoV-2 in faecal donor samples for transplantation and in asymptomatic emergency surgical admissions
Source: F1000Res. 2021 May 11;10:373. [Version 1] doi: 10.12688/f1000research.52178.1 (PMC8311808; doi:10.12688/f1000research.52178.1)
Supplement: Peer review history

# Peer review: Reviewer response for version 1

**DOI:** 10.5256/f1000research.55418.r89234

**Published:** 2021-07-21

**Authors:** James Kinross

**Affiliations:** 1 Department of Surgery and Cancer, Imperial College London, London, UK

FMT is a highly effective intervention in the treatment of CDI, and it is of significant importance that this can continue to be administered to patients during all phases of the pandemic. Moreover, the gut represents and important mediator of the inflammatory response to COVID-19 and it is a potential source of transmission. 

 This analysis has attempted to create a novel assay for the detection of SARS-Cov-2 in faecal samples based on RT-qPCR and it has validated LAMP, LamPORE and droplet digital PCR for SARS-CoV-2 RNA detection. The study has applied an in-vitro analysis and validated these data in a prospective cohort of patients attending a UK NHS hospital.

 This is a well written and well performed analysis. There is a grammatical error in the abstract conclusion. This should read (I assume..) "SARS-CoV-2 can be detected...". 

 The methodology is appropriate and well performed. The performance of the respective assays is reported. 

 The essential conclusion of this method development paper is that FMT samples could be safely tested for SARS-CoV-2 using RT-PCR or LamPORE. This adds weight to the concept that FMT services could in theory be safely re-started if these screening approaches were adopted. However, it is not possible to draw significant clinical inferences from this data set or to make comment on transmissibility of SARS-CoV-2 from faecal samples. 

 I note that some of the symptomatic patients in this cohort were treated with Remdesivir, and none of these patients had detectable virus in the samples. Those with detectable viral load had some missing treatment data, but largely had either oxygen, antibiotics and in one case steroids. Therefore it is possible that there is some selection bias.  

 The clinical validation component of this study was not powered to an endpoint, but rather served as a pilot data set to demonstrate the performance of the assays under real world conditions.

Is the work clearly and accurately presented and does it cite the current literature?

Yes

If applicable, is the statistical analysis and its interpretation appropriate?

Yes

Are all the source data underlying the results available to ensure full reproducibility?

Yes

Is the study design appropriate and is the work technically sound?

Yes

Are the conclusions drawn adequately supported by the results?

Yes

Are sufficient details of methods and analysis provided to allow replication by others?

Yes

Reviewer Expertise:

Gut microbiome, colorectal surgery and COVID19, cancer.

I confirm that I have read this submission and believe that I have an appropriate level of expertise to confirm that it is of an acceptable scientific standard.

# Peer review: Reviewer response for version 1

**DOI:** 10.5256/f1000research.55418.r89087

**Published:** 2021-07-20

**Authors:** Jonathan Landy

**Affiliations:** 1 West Hertfordshire NHS trust, Watford, UK

FMT services are essential for patients with refractory or recurrent *Clostridium difficile*. The availability of FMT services during the COVID-19 pandemic has been problematic due to concerns regarding safety and screening for SARS-COV-2 in donor stool. Although no confirmed evidence of faecal-oral transmission is available, viral shedding in the GI tract has been established up to 5 weeks after symptom onset and the potential for transmission from donor stool during the pandemic theoretically exists. There is a need to ensure safety of FMT samples during the COVID-19 pandemic. 

 This report examines preparation methods and assays for detection of SAR-CoV-2 RNA in stool that might be employed in screening of donor stool to enable safe resumption of FMT services. Due to RNA degradation lysate spiked samples were used to assess detection at varying dilutions demonstrating the validity of the assays. Furthermore, assays were confirmed in symptomatic COVID-19 patients and in a swab positive asymptomatic patient. 

 Although there may be concern regarding RNA degradation in preparation and homogenation of samples, virus can be detected in stool. However, in lower titres the assays did not detect viral RNA. This may be of concern in asymptomatic individuals with negative nasopharyngeal swabs where titres may be present, but below the limit of detection. 

 This report demonstrates the feasibility of detection of SARS-CoV-2 in screening of donor stool. However, uncertainty remains regarding the utility of this over repeated nasopharyngeal testing and exclusion of positive cases for a prolonged period and further data on current donor samples and recipients' will be important as FMT services resume.

Is the work clearly and accurately presented and does it cite the current literature?

Yes

If applicable, is the statistical analysis and its interpretation appropriate?

Not applicable

Are all the source data underlying the results available to ensure full reproducibility?

Yes

Is the study design appropriate and is the work technically sound?

Yes

Are the conclusions drawn adequately supported by the results?

Yes

Are sufficient details of methods and analysis provided to allow replication by others?

Yes

Reviewer Expertise:

FMT, microbiota, gut immunology, IBD

I confirm that I have read this submission and believe that I have an appropriate level of expertise to confirm that it is of an acceptable scientific standard.
